# Monitoring the Initiation and Kinetics of Human Dendritic Cell-Induced Polarization of Autologous Naive CD4^+^ T Cells

**DOI:** 10.1371/journal.pone.0103725

**Published:** 2014-08-21

**Authors:** Tammy Oth, Melanie C. A. Schnijderberg, Birgit L. M. G. Senden-Gijsbers, Wilfred T. V. Germeraad, Gerard M. J. Bos, Joris Vanderlocht

**Affiliations:** 1 Division of Hematology, Department of Internal Medicine, School of Oncology and Developmental Biology, Maastricht University Medical Center+, Maastricht, the Netherlands; 2 Tissue Typing Laboratory, Department of Transplantation Immunology, School of Oncology and Developmental Biology, Maastricht University Medical Center+, Maastricht, the Netherlands; New York University, United States of America

## Abstract

A crucial step in generating *de novo* immune responses is the polarization of naive cognate CD4^+^ T cells by pathogen-triggered dendritic cells (DC). In the human setting, standardized DC-dependent systems are lacking to study molecular events during the initiation of a naive CD4^+^ T cell response. We developed a TCR-restricted assay to compare different pathogen-triggered human DC for their capacities to instruct functional differentiation of autologous, naive CD4^+^ T cells. We demonstrated that this methodology can be applied to compare differently matured DC in terms of kinetics, direction, and magnitude of the naive CD4^+^ T cell response. Furthermore, we showed the applicability of this assay to study the T cell polarizing capacity of low-frequency blood-derived DC populations directly isolated *ex vivo*. This methodology for addressing APC-dependent instruction of naive CD4^+^ T cells in a human autologous setting will provide researchers with a valuable tool to gain more insight into molecular mechanisms occurring in the early phase of T cell polarization. In addition, it may also allow the study of pharmacological agents on DC-dependent T cell polarization in the human system.

## Introduction

Since the first description of two different subsets of activated murine T helper (Th) cells [1], the understanding on induction, phenotypic discrimination and functional consequences of the different Th lineages has enormously expanded. The polarization of naive CD4^+^ T cells into different Th subtypes is a tightly controlled and regulated process, which is induced by the interaction of CD4^+^ T cells with pathogen-triggered antigen-presenting cells (APC), such as dendritic cells (DC). This interaction consists of antigen-specific, co-stimulatory and polarizing signals [2], [3]. Th lineage specialization is induced according to the nature of the pathogenic insult [2], [4]–[6] and the different Th lineages are defined by the expression of various transcription factors and cytokines. Uncontrolled activation and dysregulation of this process is associated with immune-related diseases [7]–[9]. It is of crucial importance to get more insight into initiating pathways and regulating networks involved in human CD4^+^ T cell polarization to identify potential intervention points for therapy and to improve diagnostics.

Over the last decade, it has become clear that the functional differentiation of Th cells is a complex process in terms of lineages, involvement of transcription factors, plasticity and epigenetic imprinting. Whereas lineage-specifying factors, e.g. T-bet for Th1 [10], were initially considered as being both required and sufficient to determine the Th phenotype, recent research revealed their possible co-expression and interplay in one particular Th subtype [11], [12]. Moreover, the transcriptional programming during the initiation of CD4^+^ T cell responses has been shown to be imprinted only to a limited extend by these Th lineage-specifying factors and instead by cytokine-regulated environmental response factors, such as signal transducer and activator of transcription (STATs) [13], [14]. Furthermore, it has been shown that polarized Th subsets are not terminally differentiated and retain a certain degree of flexibility and plasticity; they are able to respond and adapt their phenotype to changing environmental cues [15]. These environmental changes, e.g. invasion of pathogens causing cell damage or inflammation, are sensed by DC and translated into an appropriate immune response [6]. The concentration as well as the nature of these ‘danger’ signals influence the expression of co-stimulatory molecules and the cytokine profile of DC [16]–[22] and thereby indirectly the T cell priming. It is important to study naive CD4^+^ T cell polarization in an APC-dependent manner to better understand how different pathogens induce specific Th responses and to take into account the complex network of soluble and membrane-bound factors presented by DC.

Up-to-date the majority of knowledge on Th cell differentiation was generated using murine models, such as the OT-II system or knockout models lacking specific genes involved in T cell skewing. However, murine and human immune systems display some crucial differences. This makes the extrapolation of data generated from these models in the context of human Th cell polarization challenging. Murine and human DC subsets display divergences in terms of cytokine profiles [23], pattern recognition receptor (PRR) expression patterns [24]–[29], and ligand recognition [30]. In addition, the CD4^+^ T cells show interspecies heterogeneity in terms of cytokine secretion by the different Th subtypes [31], [32] and the involvement of lineage-specifying transcription factors [33]–[35]. These divergences illustrate that the mechanisms and therapeutic interventions identified in murine models require confirmation in human *in vitro* systems before their translation into clinical trials. This need of valorization is underscored by studies revealing the possibility to cure mice but not humans with a similar treatment [36]–[38]. However, human *in vitro* assays to study the APC-dependent initiation of naive CD4^+^ T cell polarization are still limited.

Importantly, efforts were undertaken to study the kinetics of the *in vitro* programming of human naive CD4^+^ T cells using high-throughput genome-wide microarrays [39], [40]. The advantage of this approach is gaining insight into the kinetics of the individual molecular events and pathways during the differentiation of naive T cells into specific lineages, which may result in the identification of therapeutic targets; the limitation is the APC-independent setup. Even though this approach can be used as complementary method to study the involvement of single or multiple soluble factors in the initiation of a T cell response, the contribution of DC-derived contact-dependent factors is ignored. Their importance for the induction of a proper Th response has been shown [4] and thus it is important to study the early molecular events during the differentiation of naive CD4^+^ T cells in an APC-dependent manner. In current APC-dependent assays many confounders exist: medium usage, source and purity of cells, restimulation, ratio of effector:target cells, time point of measurement, culture density and the use of superantigens [4], [6], [41]–[46]. Most importantly, these current approaches do not address the initiation phase of naive DC-induced CD4^+^ T cell responses without adding supplemental environmental or blocking factors to the co-cultures. Furthermore, the monitoring of a broader range of the induced responses is limited.

We set up a system to study the initiation phase of autologous naive CD4^+^ T cell polarization in an APC-dependent and TCR-restricted manner. This system allows studying the effect of different PRR stimuli on DC-mediated direction, potency and kinetics of Th cell differentiation. It takes into account how DC-derived soluble factors interact together with co-stimulatory molecules during priming of naive CD4^+^ T cell responses without additional artificial stimulation of the co-culture, e.g. addition of Th polarizing cytokines. It allows the comparison of differently matured DC as well as different DC subsets and has the possibility to monitor the kinetics and magnitude of the lineage-specifying transcription factors of the different Th lineages and their cytokine profiles in parallel in a small-scale, serum-free set-up.

## Materials and Methods

### Generation of dendritic cells

#### Monocyte-derived DC

Leukapheresis products obtained from healthy volunteers were used to isolate the monocytes; this study was approved by the local Medical Ethics Committee of Maastricht University Medical Center, the Netherlands (MEC azM/UM; MEC 08-2-120) and written informed consent was obtained from all participating healthy volunteers. Enrichment of monocytes from leukapheresis products was achieved by counterflow centrifugal elutriation using the Elutra Cell Separation System monocyte enrichment application (Elutra, Terumo BCT Inc., Lakewood, Colorado, USA). Purity of enriched monocyte fractions was 87.9%±5.2 as assessed by flow cytometry with an average yield of 1.74×10^9^ monocytes ±0.96 per leukapheresis. Characterization of contaminating cells revealed presence of B cells, NK cells, granulocytes, and T cells. The latter counted up for 0.77%±0.22. Monocytes were frozen at 50×10^6^ cells per vial in 1 ml freeze medium: 86% autologous plasma+10% DMSO (WAK Chemie, Steinbach/Ts. Germany) +4% Glucose (50% Glucose; B. Braun Melsungen AG, Melsungen, Germany). Upon thawing, monocytes were differentiated in serum-free AIM-V medium (Invitrogen, Carlsbad, California, USA) supplemented with GM-CSF (400 U/ml; Berlex Laboratories Inc., Montville, New Jersey, USA) and IL-4 (2000 U/ml; Strathmann Biotech AG, Hamburg, Germany) at a density of 2×10^6^ cells/ml. After 7 days, iDC were harvested and processed in the DC-T cell co-culture experiments. Unless differently stated, monocyte-derived (moDC) were used.

#### Plasmacytoid DC

For the isolation of plasmacytoid DC (pDC), 500 ml of fresh heparin-anticoagulated peripheral blood was used as starting material, because of the low frequency of CD304^+^ cells in the blood, allowing the separation of up to 2.5×10^6^ cells. Blood was obtained from Sanquin blood bank Maastricht, the Netherlands (project 2000-03AZM) from healthy donors who agreed to donate their blood for research purposes and signed an informed consent. Peripheral blood mononuclear cells (PBMC) of healthy donors were isolated by density gradient centrifugation using lymphoprep (Axis-Shield PoC AS, Oslo, Norway). pDC were isolated by positive immunomagnetic cell separation using a CD304 enrichment kit (Miltenyi Biotech GmbH, Bergisch Gladbach, Germany) according to manufacturer’s instructions. Purity of enriched pDC fractions exceeded 85% as assessed by flow cytometry, with contaminating CD4^+^CD45RO^+^ cells <0.5% of all living cells. pDC were further processed in the DC-T cell co-culture experiments. The remaining PBMC fraction (flow-through) was resuspended in serum-free AIM-V medium at a concentration of 15×10^6^ cells/ml and incubated overnight at 37°C and 5% CO_2_. This suspension was used as a starting material to isolate naive CD4^+^CD45RA^+^ T cells.

### T cell isolation

Autologous total CD4^+^, CD4^+^CD45RA^+^, and CD4^+^CD45RO^+^ T cells were isolated from 500 ml of fresh heparin-anticoagulated peripheral blood. PBMC were obtained by density gradient centrifugation and further processed by negative immunomagnetic separation using a total CD4^+^ or CD4^+^CD45RO^+^ T cell isolation kit (Miltenyi Biotech) following the manufacturer’s instructions. Purities of the different T cell subsets exceeded 95%. The isolation of untouched CD4^+^CD45RA^+^ T cells was optimized as illustrated in **Figure S1**. The negative immunomagnetic separation procedure was performed twice as the isolation of these highly pure naive CD4^+^ T cells required two consecutive rounds of separation. The highest purity (≥99.8% with ≤0.1% CD4^+^CD45RO^+^) was achieved by incubating the flow-through of the first separation round a second time with the combination of antibody-mix and beads. A limitation of this second isolation procedure is the accompanied cell loss of 30–60%. The yield of isolated naive CD4^+^ T cells was donor-dependent. Autologous CD4^+^CD45RA^+^ T cells for the pDC-T cell co-cultures were isolated from the remaining fraction obtained after positive pDC selection, which has been incubated overnight at 37°C, 5% CO_2_ at 15×10^6^ cells/ml in serum-free AIM-V medium.

### T cell polarization assays

#### APC-dependent assay

iDC were matured in round bottom 96-well plates (0.2×10^5^ moDC/well or 0.1×10^5^ pDC/well) with different maturation cocktails: PGE_2_ (18 µg/ml; Sigma-Aldrich, St. Louis, Missouri, USA) and TNF-α (1000 U/ml; Biosource International, Camarillo, California, USA); FMKp (10 µg/ml; Pierre Fabre Laboratories, Boulogne-Billancourt, France) and IFN-γ (500 U/ml; R&D systems, Minneapolis, Minnesota, USA); LPS (200 U/ml; USP, Rockville, Maryland, USA) and IFN-γ (1000 U/ml); HKLM (10^9^ cells/ml; Invivogen, Toulouse, France) and IFN-γ (500 U/ml); or ODN2216 (10 µM/ml; Invivogen). For moDC all cocktails were supplemented with IL-4 (500 U/ml) and GM-CSF (500 U/ml) and for pDC with IL-3 (100 ng/ml; Miltenyi Biotech). The optimal moDC-T cell co-culture ratios were determined by culturing increasing numbers of 24 h-matured FMKp/IFN-γ-moDC (0.1, 0.2, 0.5×10^5^) with 0.5×10^5^ autologous naive CD4^+^ T cells (**Figure S2**). To this end, 24 h-matured DC were harvested, washed and transferred at different ratios into round bottom 96-well plates together with an equal amount of pooled supernatant of 24 h-matured DC. Because of their higher T cell stimulatory capacity shown in a pilot experiment (data not shown), we co-cultured pDC and naive T cells at a ratio of 1∶5. In the condition with ‘washed FMKp/IFN-γ-DC’, the maturation stimuli were removed 6 h after the induction of maturation by washing the DC three times with medium and adding 50 µl of fresh serum-free medium. After 24 h of maturation, DC were loaded with 50 µg/ml of a pan HLA-DR binding peptide (PADRE; AGVAAWTLKAAA) that binds all groups of HLA-DR variants and leads to a polyclonal TCR-restricted T cell stimulation [47]. After 1 h of incubation, 0.5×10^5^ autologous T cells were added in 25 µl to the different wells. As a negative control, T cells were cultured in serum-free medium in the absence of DC. During the 7-day co-culture, samples were collected by harvesting each day individual wells of the different conditions. Supernatant was harvested and frozen for cytokine determination, whereas the cell pellets were lysed in RLT buffer (Qiagen, Alameda, California, USA) containing 1% 2-mercaptoethanol (Sigma-Aldrich) and stored at −80°C for gene expression analyses. Furthermore, supplementary wells were cultured to study surface activation markers, as well as to determine the presence of intracellular IFN-γ on day 7 of the co-culture. GolgiStop and GolgiPlug (both BD Biosciences, Franklin Lakes, New Jersey, USA) were added to the latter wells and cells were incubated overnight in absence or presence of PMA (0.25 µg/ml) and ionomycin (0.5 µg/ml; both Sigma-Aldrich).

#### APC-independent assay

Round bottom 96-well plates were coated overnight with 0.25 µg/ml anti-CD3 (clone 2G3, kind gift from Prof. Dr. N. Hellings, Biomedical Research Institute, Diepenbeek, Belgium). After extensive washing of the plates, 0.5×10^5^ pure CD4^+^CD45RA^+^ T cells were cultured in serum-free medium in absence or presence of DC supernatant. This supernatant was generated by maturing iDC at a density of 5×10^5^/ml for 6 h in the presence of IL-4 (500 U/ml), GM-CSF (400 U/ml), IFN-γ (500 U/ml) and FMKp (10 µg/ml). After the induction of maturation, adherent cells were washed three times to remove the maturation stimuli and fresh medium was added. After a total of 48 h, DC supernatants were collected. During the 5-day co-culture, each day the culture supernatant was harvested and frozen at −20°C for cytokine determination. Cells were harvested and lysed in RLT buffer supplemented with 1% 2-mercaptoethanol and frozen at −80°C for gene expression analysis.

### Flow cytometry

All flow cytometry antibodies used to determine DC and T cell purities as well as Th cell surface marker expression were purchased from BD Biosciences, except for BDCA-2 (Miltenyi Biotech). Antibodies were used at the proper concentration either as fluorescein isothiocyanate, phycoerythrin, peridinin chlorophyll protein, allophyocyanin, allophyocyanin-H7, Horizon 450, Horizon 500, Alexa Fluor 488 or Pe-Cy7. Intracellular IFN-γ staining was performed as described previously [48]. Analyses were performed with BD FACS Canto II and analyzed by BD FACSDiva Software v6.1.2 (BD Biosciences).

### qPCR

Total cellular RNA was isolated from lysed T cells using RNeasy Mini kit (Qiagen) according to manufacturer’s instructions. Genomic DNA was removed by DNase I (Invitrogen) treatment followed by reverse transcription using random hexamer primers (Invitrogen) and Superscript III Reverse Transcriptase (Invitrogen) according to the standard procedures. Primers for quantitative PCR analysis were generated using primer-BLAST software (NCBI). All primers were selected to span exon-exon boundaries with a maximal amplification length of 300 bp. Primer specificity was tested by melting curve analyses, product size confirmation by gel-based PCR analyses and bidirectional sequencing of amplified products after subcloning in plasmids (Topo TA cloning, Invitrogen). Relative quantification was done by generating a standard curve of a reference sample (cDNA of total T cell fraction). Real-time PCR was done with SYBR green detection (SensiMix SYBR & Fluorescein Kit, both from Bioline Reagents Ltd., London, United Kingdom) using an iCycler iQ (Biorad Laboratories Inc., Hercules, California, USA) and primers (10 pmol) specific for the different Th lineages (listed in **Table S1**). The PCR program consisted of 10 min initial heating at 95°C (*hot start* polymerase), followed by 35 cycles amplification (30 s at 95°C, 20 s at the optimized annealing temperature and 20 s at 72°C) and a final heating up to 92°C (increasing 0.5°C/7 s) for the generation of a melting curve. Each data point of the graphs was generated by determining expression of the gene of interest on the different days in duplicate and normalizing these data to the corresponding expression of CD3ε used as housekeeping gene in our assay.

### helper cell cytokine detection

Quantification of Th-specific cytokines (IFN-γ, IL-4, IL-5, IL-13, IL-17, IL-10) in the supernatant of the DC-T cell co-cultures on day 1, 3, 5 and 7 (day 1, 3 and 5 for the anti-CD3 culture) was performed by CBA flex set assay (BD Biosciences) according to the manufacturer’s instructions. Measurements were performed with BD FACS Canto II and analyzed by BD FACSDiva Software v6.1.2 and FCAP array analysis software (version 1.0.1; Soft Flow Inc., St. Louis Park, Minnesota, USA).

### Statistical analyses

Statistical analyzes were performed by Wilcoxon matched pairs test and the correlation was tested by nonparametric Spearman correlation; *P* values<0.05 were regarded as significant. Data was analyzed using Prism Software (version 6; GraphPad Software, San Diego, California, USA).

## Results

### Development and set-up of an APC-dependent, TCR-restricted naive CD4^+^ T cell polarization assay

**Figure 1:**
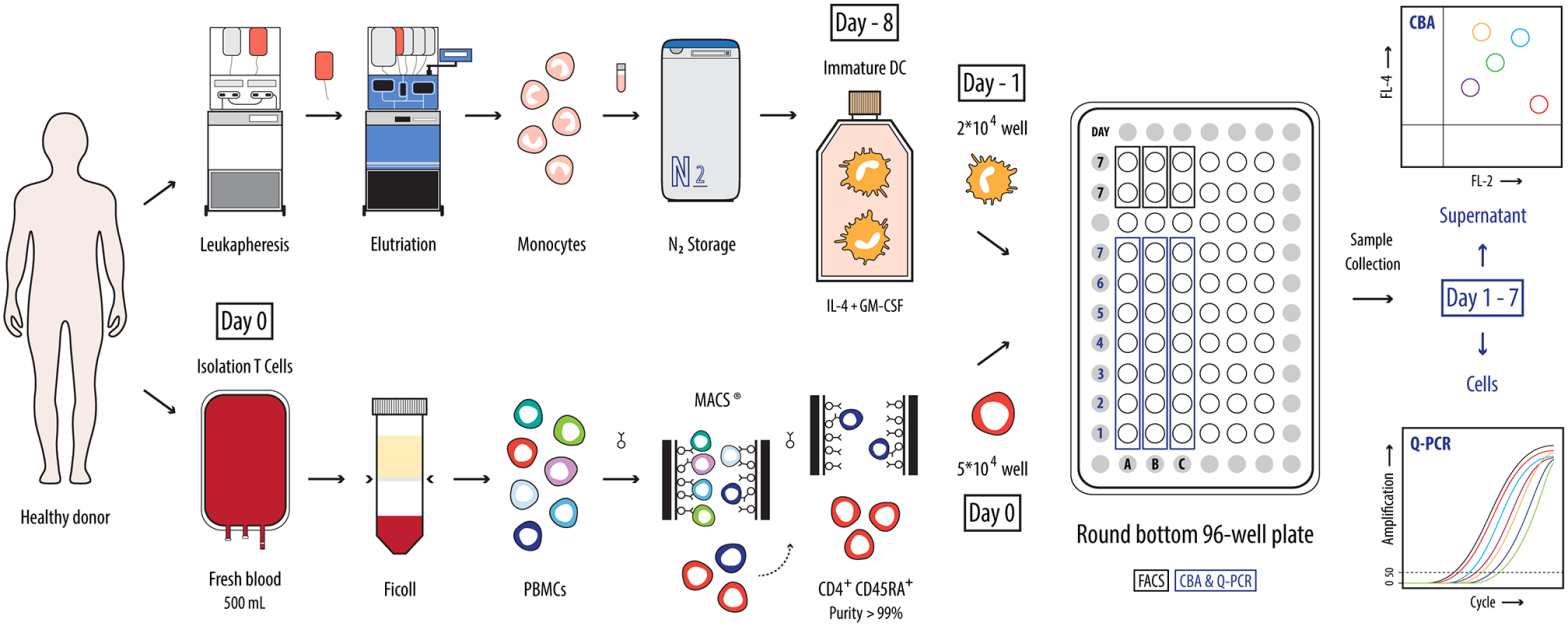
Schematic representation of the APC-dependent assay to monitor DC-induced naive CD4^+^ T cell polarization. Monocytes were isolated from leukapheresis products obtained from healthy volunteers by counter flow centrifugal elutriation. These enriched monocytes (purity 87.9%±5.2) were frozen and upon thawing differentiated into iDC using IL-4 and GM-CSF. After 7 days, iDC were harvested and plated in a round bottom 96-well plate (2×10^4^ cells/well) in presence of maturation stimuli (A–C). The following day, autologous naive CD4^+^ T cells were isolated from fresh PBMC obtained from 500 ml of blood by negative immunomagnetic separation (purity of 99.9% of total CD4^+^ T cells) and 5×10^4^ T cells were added to the different wells, preceded by a 1-hour incubation with PADRE, a pan HLA-DR-restricted peptide. The co-culture was maintained over a period of 7 days and each day supernatant and cells of a single well of each condition were harvested to analyze the secretion of Th-specific cytokines (CBA) and the expression of Th-related transcription factors and cytokines (qPCR).

We set out to develop a TCR-restricted, human autologous assay which allows the comparison of the CD4^+^ T cell polarizing capacity of differently matured DC using primary cells. In order to develop such an APC-dependent system a number of prerequisites need to be considered. These include e.g. the selection of an appropriate polyclonal antigen, isolation of pure cells and determining appropriate cell ratios. A schematic overview of the optimized assay is represented in **Figure 1**. In the following sections more details about the assay development will be provided.

#### Antigen selection

The human precursor frequencies of epitope-specific naive CD4^+^ T cells, counting up to at most 100 cells/10^6^ naive T cells [49], limited the development of small-scale 96-well format assay for the comparison of human naive cognate CD4^+^ T cell responses. To circumvent this, we used a pan HLA-DR-restricted peptide, named PADRE, which polyclonaly activates a subset of T cells [47]. PADRE differs from superantigens as it binds inside the peptide-binding groove of HLA-DR. PADRE has the advantage of binding to all major groups of HLA-DR-restricted variants, which makes it a TCR-restricted peptide for the study of CD4^+^ T cell responses in the majority of individuals.

#### Co-culture ratios

To determine the optimal DC:T cell ratio, we co-cultured different concentrations of DC with a constant number of CD4^+^CD45RA^+^ T cells (5×10^4^/well). To exclude the influence of different concentrations of DC-derived factors on the initiation of the T cell response, DC were washed after 24 h of maturation prior to their addition to the co-culture. Additionally, all the co-cultures were supplemented with the same amount of pooled 24 h-matured DC-derived supernatant. Based on these experiments (**Figure S2**), we selected a DC:T cell ratio of 2∶5 to perform the co-culture experiments, as the amount of T cell-derived IFN-γ in these Th1 polarizing conditions was shown to be the highest.

#### Set-up of the co-culture

Summarizing, the ultimate setup of the assay is as follows (**Figure 1**): to maximize the priming efficiency of DC [43], iDC were matured in a round bottom 96-well plate in serum-free AIM-V supplemented with different maturation stimuli. The next day, naive CD4^+^CD45RA^+^ T cells were isolated from fresh PBMC and added to the 24 h-matured DC preceding a 1 h-incubation with PADRE. During a 7-day co-culture, each day supernatant and cells were harvested from a single well to determine Th cytokines and Th lineage-specifying transcription factors by CBA and qPCR, respectively.

### Monitoring the initiation of Th1 responses

In our search for a DC maturation stimulus capable of polarizing naive CD4^+^ T cells towards a Th1 phenotype, we used a combination of FMKp and IFN-γ as maturation cocktail. This selection was based on previous demonstrations that FMKp/IFN-γ-matured DC were able to produce high IL-12 levels [48], [50], [51], a cytokine which is crucial for Th1 responses [52], [53]. Furthermore, these DC were shown to be potent inducers of CTL responses [50].

**Figure 2:**
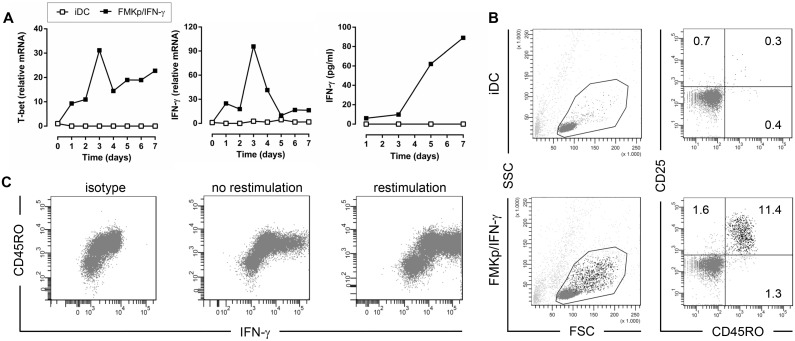
FMKp/IFN-γ-matured DC polarize a subpopulation of naive T cells into the Th1 lineage. iDC or FMKp/IFN-γ-matured DC were pulsed with PADRE for 1 h before the addition of autologous CD4^+^CD45RA^+^ T cells to the 7-day co-culture. (A) Expression of T-bet and IFN-γ and IFN-γ production of naive CD4^+^ T cells co-cultured with iDC (□) or FMKp/IFN-γ-matured DC (▪) are compared. CD3ε was used as housekeeping gene and the expression data were normalized to the relative mRNA content of naive T cells on day 0. IFN-γ production by naive T cells co-cultured with differently matured DC was determined in the supernatant of the co-culture on day 1, 3, 5 and 7 by CBA. Graphs are representative of 11 independent experiments. (B) On day 7, T cells were stained for expression of CD45RO and CD25 and analyzed by flow cytometry. Cells are gated in FSC/SSC on lymphocyte gate, excluding dead cells and doublets (light gray dots), and selected for CD4^+^ cells (dark gray and black dots). Percentages of CD45RO and CD25 positive populations are indicated in the plots. Dot plots are representative of 5 independent experiments. (C) On day 6 of the co-culture, GolgiPlug and GolgiStop were added to T cells with or without PMA/ionomycin restimulation. The next day, intracellular IFN-γ staining was performed and cells were analysed by flow cytometry. Representative dot plots of CD45RO and IFN-γ expression of CD4^+^ T cells co-cultured with FMKp/IFN-γ-matured DC are shown. Data are representative of 5 independent experiments.

To study the initiation of a Th1 response, we compared the polarizing capacities of iDC and FMKp/IFN-γ-matured DC (**Figure 2**). FMKp/IFN-γ-matured DC induced the lineage-specifying transcription factor T-bet (gene name *TBX21*) and IFN-γ mRNA levels already after 24 hours and were peaking on day 3 (30 resp. 90-fold increase in mRNA levels compared with T cells on day 0). IFN-γ mRNA induction was associated with IFN-γ cytokine production as evidenced by CBA. T cells co-cultured with iDC only showed minor upregulation of T-bet levels (up to 5-fold) and no upregulation of IFN-γ levels (**Figure 2A**). Additionally, T cells of the two co-cultures were stained on day 7 for different activation markers and analyzed by flow cytometry. Of the T cells co-cultured with FMKp/IFN-γ-matured DC, 11.4% were double positive for CD25 and CD45RO (**Figure 2B**) compared with 0.3% in the co-culture with iDC. In addition, these double positive cells were enlarged in the FSC/SSC compared with the double negative ones.

We performed an intracellular IFN-γ staining to identify whether or not the amount of IFN-γ secreted on day 7 of the co-culture was derived from a few high-producing cells. Protein transport inhibitors were added on day 6 of the co-culture and the cells were incubated overnight. To enhance the cytokine secretion of all Th1-committed cells, we performed an additional intracellular cytokine staining on restimulated cells. The percentage of IFN-γ^+^-T cells was higher in the co-culture of FMKp/IFN-γ-matured DC compared with iDC (data not shown). This corresponds to the higher total IFN-γ levels detected in the supernatant of this condition. Even though restimulation of the cells for 16 h with PMA/ionomycin was not required to detect the accumulation of intracellular IFN-γ in CD4^+^ T cells, more Th1-committed CD4^+^ T cells were detected in the restimulated condition on day 7 of the co-culture (**Figure 2C**). This indicates that restimulation is required for estimating the total size of the Th1-committed subpopulation.

Taken together, these data indicate that PADRE-loaded FMKp/IFN-γ-matured moDC are capable of polarizing a subset of naive CD4^+^ T cells into the Th1 lineage as evidenced by their upregulation of T-bet and IFN-γ mRNA levels, their expression of CD25 and CD45RO, and the secretion of IFN-γ.

#### Th1 polarization is not a direct effect of the maturation stimuli on T cells

**Figure 3:**
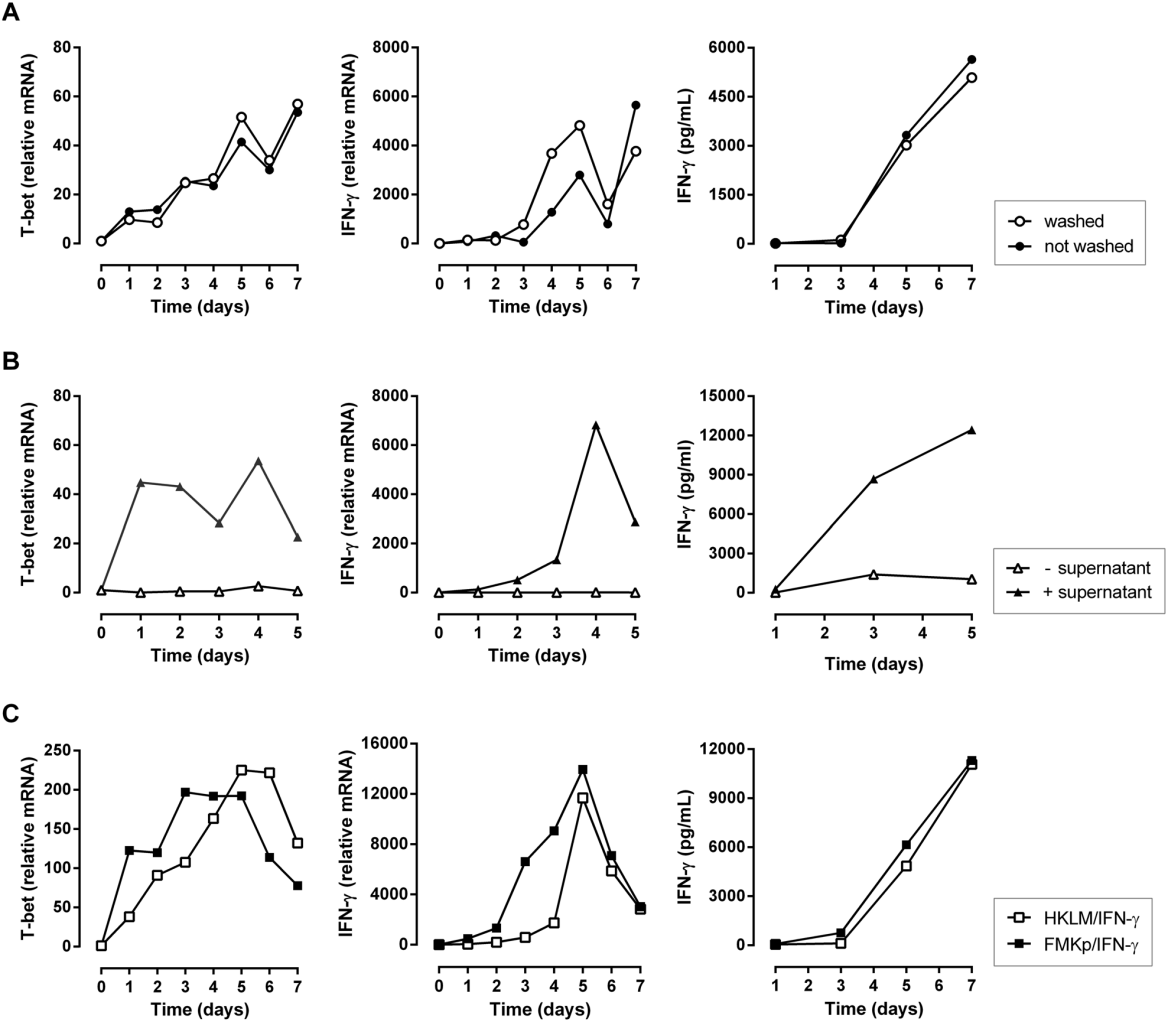
Dependency of DC-induced Th1 polarization on DC-derived soluble factors. (A) FMKp/IFN-γ-matured DC were either extensively washed (○) or not (•) 6 h after induction of maturation and co-cultured for 7 days with autologous CD4^+^CD45RA^+^ T cells. Expression of T-bet and IFN-γ and total IFN-γ production are shown. Graphs are representative of 5 independent experiments. (B) APC-independent assay using immobilized anti-CD3 allows studying the influence of DC-derived soluble factors on T cell polarization. 5×10^4^ cells CD4^+^CD45RA^+^ T cells were cultured for 5 days without (Δ) or with (▴) washed FMKp/IFN-γ-matured DC-derived supernatant in presence of plate-bound α-CD3 (0.25 µg/ml) in a round bottom 96-well plate. Transcriptional induction of T-bet and IFN-γ and secretion of IFN-γ were determined. Data shown are representative data of 3 independent experiments. (C) FMKp/IFN-γ- and HKLM/IFN-γ-matured DC induce Th1 polarization. iDC were matured with HKLM/IFN-γ (□) or FMKp/IFN-γ (▪) and co-cultured with CD4^+^CD45RA^+^ T cells. Expression of T-bet and IFN-γ and total IFN-γ production are shown. Data are representative of 4 independent experiments.

We performed washing experiments to exclude that T cells are polarized in response to the DC maturation stimuli instead of their interaction with matured DC and their released cytokines. Previously, we and others [6], [48], [54] have shown that an imprinting of the DC maturation of 6 h is sufficient to trigger the DC maturation program. We compared CD4^+^ T cell polarizing capacity of FMKp/IFN-γ-matured DC incubated for 24 h with maturation stimuli with those of 6 h after induction of maturation extensively washed FMKp/IFN-γ-matured DC. Both washed and non-washed FMKp/IFN-γ-matured DC induced the expression of T-bet and IFN-γ following the same pattern, as well as comparable levels of IFN-γ after 7 days of co-culture (**Figure 3A**). These data confirm that the Th1 polarization seen by co-culturing FMKp/IFN-γ matured DC with naive CD4^+^ T cells is APC-dependent and is not a consequence of direct influence of the DC maturation stimuli on the T cells.

#### Effect of FMKp/IFNγ-derived cytokines on Th1 polarization

To further investigate the contribution and importance of the DC-derived soluble factors on T cell polarization, an APC-independent approach can be used as complementary tool to study their influence independently of the co-stimulatory and other membrane-bound factors. To this end, we cultured naive CD4^+^ T cells for 5 days on immobilized anti-CD3 in the absence or presence of FMKp/IFN-γ-DC-derived supernatant. To compare results obtained by this assay with our APC-dependent assay and to avoid the direct polarizing effect of the immobilized anti-CD3, we titrated down anti-CD3 to such an extent that it gave a similar proliferative response as observed in the APC-dependent assay (data not shown). The influence of DC-derived soluble factors on naive T cells was tested with supernatant derived from 24 h-matured FMKp/IFN-γ-DC, which were washed extensively 6 h after induction of maturation. As shown in **Figure 3B**, in the presence of FMKp/IFN-γ-DC-derived supernatant the expression of both T-bet and IFN-γ was induced and IFN-γ was detected in the culture supernatant as evidenced by CBA. These data illustrate that APC-independent systems are complementary to our APC-dependent system allowing the discovery of the causative factors without interference of contact-dependent stimuli (e.g. co-stimulatory molecules).

#### Commercially available DC maturation stimuli inducing Th1 polarization

To investigate whether or not a commercially available DC maturation stimulus shows similar effects on the T cell polarization as FMKp, we screened different PRR triggers in combination with IFN-γ (data not shown). Among all the triggers we tested, HKLM/IFN-γ-matured DC had the most potent capacity to induce Th1 polarization. As shown in **Figure 3C**, a similar expression pattern of T-bet and IFN-γ was observed, with FMKp/IFN-γ-DC leading to faster induction of these factors in the T cells. The level of IFN-γ secretion over time of the co-culture was not significantly different.

#### Influence of memory CD4^+^ T cell contamination

**Figure 4:**
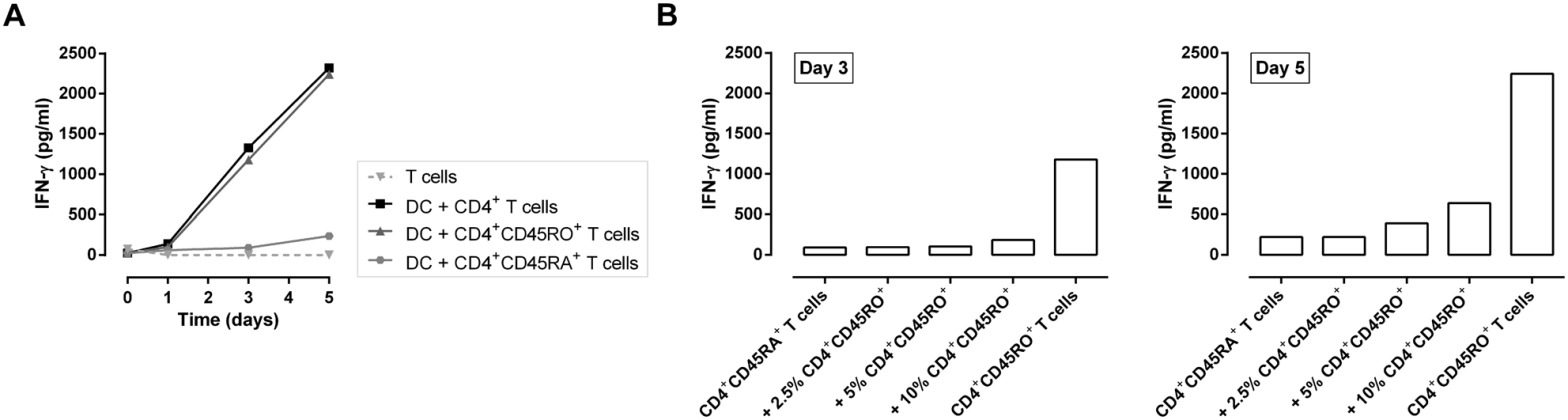
Presence of CD4^+^CD45RO^+^ T cells in the DC-CD4^+^CD45RA^+^ T cell co-culture influences Th1 read-out parameters. (A) CD4^+^, CD4^+^CD45RO^+^, CD4^+^CD45RA^+^ populations have been isolated by negative immunomagnetic separation from freshly isolated PBMC. IFN-γ production of total CD4^+^ (black square), CD4^+^CD45RA^+^ (light gray circle), and CD4^+^CD45RO^+^ (dark gray triangle) T cells co-cultured with FMKp/IFN-γ-matured DC for 7 days as measured by CBA. (B) Contribution of contaminating CD4^+^CD45RO^+^ T cells (2.5, 5 or 10%) to CD4^+^CD45RA^+^-derived IFN-γ-production compared with pure (>99.9%) CD4^+^CD45RA^+^ T cell populations. Data shown are representative of 2 independent experiments.

A disadvantage of using an HLA-DR-restricted polyclonal peptide in this assay is the activation of memory CD4^+^ T cells. They do not require a differentiation phase and thereby readily produce cytokines upon TCR-triggering. To evaluate to what extend the purity of naive CD4^+^ T cells influences our read-out parameters, we first compared Th1-inducing potential of different CD4^+^ T cell fractions. The different CD4^+^ T cell fractions – total CD4^+^, CD4^+^CD45RA^+^ and CD4^+^CD45RO^+^ T cells – were co-cultured for 5 days with FMKp/IFN-γ-matured, PADRE-pulsed DC and supernatants were harvested each day. The purities of the different CD4^+^ T cell fractions were analyzed with flow cytometry (**Figure S3**). On day 5 of the co-culture, memory CD4^+^CD45RO^+^ T cells produced as much IFN-γ as the total CD4^+^ T cell fraction; whereas naive CD4^+^CD45RA^+^ T cells produced 10 times less IFN-γ after 5 days of co-culture (**Figure 4A**).

Compared with a naive response, the magnitude of the cytokine response is 10-fold increased for the memory CD4^+^ T cells and they are the main contributors of IFN-γ secretion after 5 days of co-culture with moDC. We performed a spiking experiment to evaluate to what extend a contamination with memory T cells during the co-culture with CD4^+^CD45RA^+^ will influence the read-out parameters of our PADRE-peptide-based assay. Increasing numbers of CD4^+^CD45RO^+^ cells were added to a DC/CD4^+^CD45RA^+^ co-culture (up to 10% memory spike) and IFN-γ secretion on day 3 and day 5 of the co-culture was determined. On day 3 no differences between pure CD4^+^CD45RA^+^ co-culture and up to 5% spiking with CD4^+^CD45RO^+^ were observed, whereas the IFN-γ production of the condition with 10% CD4^+^CD45RO^+^ cells was twice as high compared with the other conditions (**Figure 4B**). On day 5 a 2-fold difference in IFN-γ production was detectable with 5% spiking and even 3-fold with 10% spiking compared with the pure CD4^+^CD45RA^+^ fraction. Taking into account that with our procedure it is possible to isolate highly pure CD4^+^CD45RA^+^ T cells (**Figure S1**) and that the contamination with CD4^+^CD45RO^+^ cells is below 0.1%, the read-out parameters of our assay are only to a minute extend biased by memory CD4^+^ T cell contamination.

### Differential T cell polarizing capacity of DC

#### Induction of Th1 and Th2 polarization by moDC

**Figure 5:**
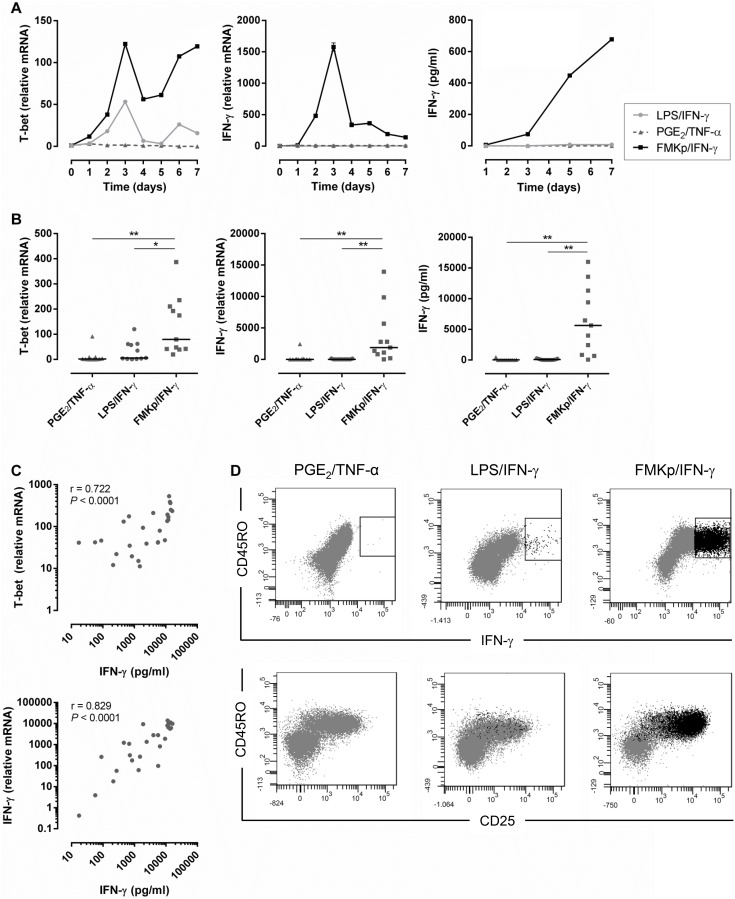
Differential Th1 polarizing capacity of differently matured DC. Naive CD4^+^ T cells were co-cultured for 7 days with PGE_2_/TNF-α (dark gray triangle), LPS/IFN-γ (light gray circle) or FMKp/IFN-γ-(black square) matured DC. (A) Expression of T-bet and IFN-γ and production of IFN-γ were monitored. Graphs are representative of 11 independent experiments. (B) Comparison of the expression of T-bet and IFN-γ on day 5 and IFN-γ production on day 7 of differently matured DC cultured with naive CD4^+^ T cells. 11 independent experiments and their median levels are shown. Wilcoxon signed-rank test significance **P≤0.01, ***P<0.001 (C) Correlation between T-bet and IFN-γ expression on day 5 and IFN-γ secretion on day 7 of FMKp/IFN-γ-matured DC in co-culture with naive CD4^+^ T cells. Nonparametric Spearman correlation test significance indicated in the graphs. (D) Expression of CD45RO, CD25, and intracellular IFN-γ of naive CD4^+^ T cells cultured for 7 days with differently matured DC as indicated above the graphs. On day 6 GolgiPlug and GolgiStop were added to the co-culture and the staining was performed on day 7. Cells shown in the plots represent living singlet cells gated on CD3^+^CD4^+^ (dark gray population). T cells positive for IFN-γ are shown in black. Dot plots are representative graphs of 3 independent experiments.

The current system allows the comparison of the T cell polarizing capacity of differently matured DC in terms of kinetics and magnitude of Th1 responses. We show the example of the capacity of 3 different (pre-) clinical DC maturation cocktails - PGE_2_/TNF-α, LPS/IFN-γ or FMKp/IFN-γ-used for the generation of *ex vivo* matured DC for DC-based vaccines and their capacity to polarize naive CD4^+^ T cells into Th1 cells (**Figure 5**). Both, in T cells co-cultured with LPS/IFN-γ and FMKp/IFN-γ-matured DC, the mRNA levels of T-bet were induced over time, being higher in the FMKp/IFN-γ-DC co-culture. mRNA levels and secretion of IFN-γ could only be detected in the co-cultures of T cells with FMKp/IFN-γ-matured DC (**Figure 5A**). In **Figure** **5B** the average expression of T-bet and IFN-γ on day 5 of the co-cultures of these three different conditions as well as the total IFN-γ production detected in the supernatant of the co-cultures on day 7 are combined. In line with the graphs of **Figure 5A**, both T cells cultured with LPS/IFN-γ and FMKp/IFN-γ-matured DC showed increased levels of T-bet (up-regulated median levels of 5.6 vs. 79.5 compared with T cells alone), but the mRNA levels and secretion of IFN-γ was limited to naive T cells cultured with FMKp/IFN-γ-matured DC. There was a significant correlation of both T-bet and IFN-γ mRNA levels on day 5 of the FMKp/IFN-γ-mDC co-cultured T cells with the accumulated IFN-γ secretion on day 7 (**Figure 5C**). Additionally, T cells of the different conditions were stained for CD45RO and CD25 as well as for intracellular IFN-γ. In all three conditions T cells up-regulated the expression of CD25 and CD45RO upon the ‘antigen’-encounter. Only in the FMKp/IFN-γ conditions, part of these cells was also positive for IFN-γ, which corresponds to data shown in **Figure 5A,B**.

**Figure 6:**
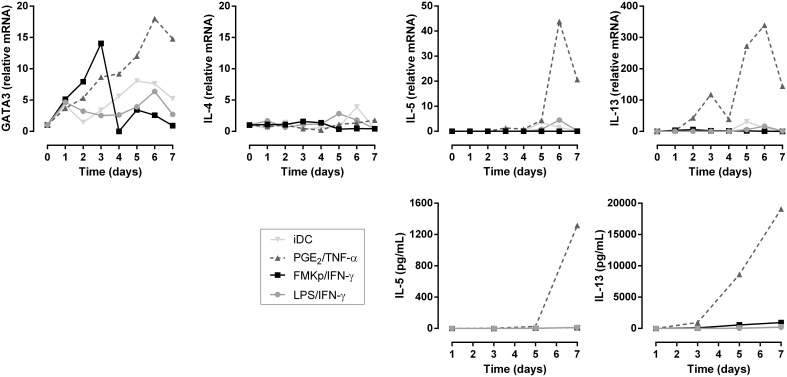
Differential Th2 polarizing capacities of differently matured moDC. Naive CD4^+^ T cells were co-cultured for 7 days with PGE_2_/TNF-α (dark gray triangle), LPS/IFN-γ (light gray circle) or FMKp/IFN-γ (black square) matured DC. Expression of GATA3, IL-4, IL-5 and IL-13 and production of IL-5 and IL-13 were monitored. IL-5 and IL-13 protein production by naive T cells co-cultured with differently matured DC was determined in the supernatant of the co-culture. Graphs are representative of 5 independent experiments.

In parallel, we studied the capacity of these differently matured DC to induce Th2 polarization, as illustrated in **Figure 6**. In all the DC-T cell co-cultures, a transient up-regulation of GATA3 mRNA levels, the lineage-specifying transcription factor of Th2 cells, was observed. Only PGE_2_/TNF-α-DC led to a constantly increasing expression of GATA3 in the T cells. In addition, the expression of IL-4, IL-5, and IL-13, the signature cytokines of the Th2 lineage, was monitored. IL-5 and IL-13 expression was only detectable in the PGE_2_/TNF-α condition, which paralleled the cytokine secretion profile; only T cells of the PGE_2_/TNF-α condition secreted IL-5 and IL-13. This is in line with our previous demonstration of CRTH2^+^CD4^+^ T cells in a co-culture of PGE_2_/TNF-α-matured DC in a total T cell pool [50]. IL-4 was not expressed nor secreted in any of the conditions. Thus, PGE_2_/TNF-α-matured DC can be used as a positive control for IL-5/IL-13-secreting Th2 cells. Unexpectedly, the Th1-inducing FMKp/IFN-γ-DC induced a transient expression of GATA3, but did not lead to any secretion of Th2 cytokines, supporting the recently published studies about co-expression of different transcription factors [11], [12].

#### Induction of Th1 polarization by pDC

**Figure 7:**
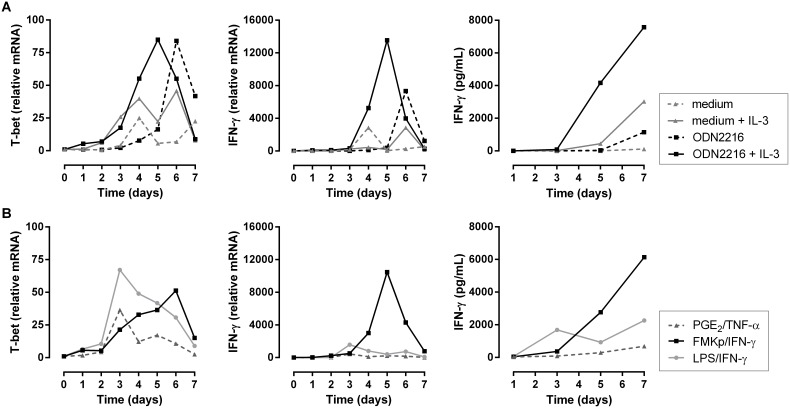
Differential Th1 polarizing capacity of differently matured plasmacytoid DC. pDC, isolated from fresh blood, were stimulated overnight with different cocktails and 24 h later autologous naive CD4^+^ T cells were added and the expression of T-bet and IFN-γ and secretion of IFN-γ were monitored during 7 days. (A) pDC were stimulated overnight with IL-3 (full gray line), ODN2216 (dashed black line) or with a combination of both (full black line). (B) Comparison of the capacity of differently matured pDC to induce Th1 polarization. pDC were incubated with PGE_2_/TNF-α (dark gray triangle), LPS/IFN-γ (light gray circle) or FMKp/IFN-γ (black square) cocktail in the presence of IL-3. Representative data from 2 independent experiments are shown.

To investigate whether or not the small-scale setup of the assay allows the study of a more infrequent blood DC subset, we studied the applicability of this system to plasmacytoid DC (pDC), which is illustrated in **Figure 7**. Unstimulated and ODN2216-triggered pDC, a microbial stimulus known to trigger pDC via TLR9 [55], were co-cultured with naive T cells during 7 days in absence or presence of IL-3. Naive CD4^+^ T cells co-cultured with unstimulated pDC up-regulated T-bet and IFN-γ mRNA and produced minor levels of IFN-γ (**Figure 7A**). IL-3 stimulation of pDC increased the magnitude of T-bet expression, induced faster kinetics and led to substantially higher levels of IFN-γ secretion (117 vs. 3026 pg/ml on day 7). T cells cultured with ODN2216-stimulated pDC, showed a delayed but overall increased T-bet expression compared with pDC alone or pDC + IL-3. The combination of ODN2216 and IL-3 stimulated pDC led to faster T-bet expression and both a faster and a higher IFN-γ expression, which was also reflected on the cytokine level. These data illustrate that IL-3 is not only an important survival factor for pDC [56], but it also influences the kinetics and the magnitude of pDC-induced T cell differentiation. ODN2216-stimulated pDC can be used as a positive control to study the effect of differently stimulated pDC on naive CD4^+^ T cell polarization.

To investigate whether or not different DC subsets translate a challenge by a particular PRR trigger into similar Th responses, we tested in the same experiment the influence of differently matured pDC on naive CD4^+^ T cell polarization (**Figure 7B**), comparing the maturation cocktails described in **Figure 5** in the presence of IL-3. Naive T cells co-cultured with these three differently stimulated pDC all expressed T-bet. IFN-γ was mainly expressed by T cells in culture with FMKp/IFN-γ-pDC, which was also reflected by the highest IFN-γ levels produced over 7 days. Even though LPS/IFN-γ-pDC showed the fastest kinetics, FMKp/IFN-γ-pDC showed 3-fold higher IFN-γ levels and even 10-fold compared with PGE_2_/TNF-α pDC on day 7 of the co-culture.

We illustrated that this APC-dependent system allows the comparison of the T cell polarization capacity of differently matured DC in terms of kinetics, magnitude and direction. Moreover, the system can be applied for functional studies with multiple DC subsets.

## Discussion

In terms of human T cell differentiation, fundamental tools to study the initiation of APC-dependent naive CD4^+^ T cell polarization are lacking. However, a dysregulation in human CD4^+^ T cell polarization is associated with a variety of diseases, such as cancer, autoimmunity, and allergies [7], [9]. Due to interspecies discrepancies, data generated from murine models on e.g. the positive effect of medication on T cell-mediated diseases awaits confirmation in human *in vitro* assays. We have described in this paper a standardized methodology to study the priming of human naive CD4^+^ T cells by differently matured DC. This *in vitro* system consists of a multi-faceted read-out tool that allows the monitoring of transcriptional events associated with T cell polarization and matching them to a Th cytokine secretion profile using a protein-multiplexing platform.

Inherent to the aim of setting up a small-scale (96-well format) assay to study CD4^+^ T cell polarization in the human system is that it does not allow antigen-specificity, because of the low precursor frequencies of epitope-specific naive CD4^+^ T cells in humans [49]. As a consequence the assay is performed in an antigen-independent but TCR-restricted manner using the HLA-DR-restricted peptide PADRE. In initial experiments, we observed that memory CD4^+^ T cells were rapidly activated in this system and that they secreted cytokines upon TCR-triggering without the need of a programming phase. A memory contamination of more than 5% in the DC-T cell co-culture contributed to at least 50% of the Th1-derived IFN-γ secretion after 5 days. In addition, memory contamination induced a faster response compared with a co-culture of highly pure naive CD4^+^ T cells (data not shown), indicating the importance of using pure naive CD4^+^ T cells in a polyclonally activated system. We have shown that these highly pure CD4^+^CD45RA^+^ T cells (>99.9% CD4^+^CD45RA^+^ and <0.1% CD4^+^CD45RO^+^) can be obtained using a simple immunomagnetic bead isolation kit without the need of a high-end flow-sorting methodology confirming previous findings [57].

Most existing assays aim to demonstrate the ultimate fate of the polarized T cell and not the initial priming phase of a naive CD4^+^ T cell response. Moser et al. (2010) introduced a DC-based system to study the *in vitro* priming of naive T cell responses aiming to predict vaccine efficacies. Even though this assay has the advantage of monitoring antigen-specific T cell responses, the co-culture period over 14 days with restimulation by fresh antigen-pulsed DC only detects late stage responses of the naive CD4^+^ T cell polarization. However, it is possible that differently matured DC do not differ in the magnitude nor in the direction of the induced T cell response but that they lead to a faster induction. This knowledge can be of importance in pharmacological studies and may create valuable insight for designing vaccines. With our system, we provide a complementary tool to study the acute phase and evaluate the kinetics during the initiation of a naive CD4^+^ T cell response.

The vast majority of *in vitro* assays studying human CD4^+^ T cell polarization in an APC-dependent manner focus on moDC. An important disadvantage of this DC population is that these cells show more similarity with monocytes than with human subsets of lymphoid-tissue-resident DC: pDC and cDC (BDCA1^+^ and BDCA3^+^) [23]. Nevertheless, it is generally accepted that moDC are good surrogates to study inflammatory DC and we optimized our assay using moDC as source of APC. In addition, we demonstrated that because of the small-scale assay set-up, our findings can be translated to other *in vivo* and low-frequent blood-derived DC subsets. Out of 500 ml fresh blood we could obtain between 1–2.5×10^6^ pDC, which allows studying the capacity of at least 14 differently stimulated pDC to prime CD4^+^ T cells. This opens new perspectives to study the human counterparts of the mouse pDC, CD8α^+^ cDC and CD11b^+^ cDC despite their very low blood frequencies. In addition, because of the low-scale set-up of this assay, we foresee that this co-culture system may also be applied to study primary tissue-resident DC subsets.

Previous studies focusing on the differentiation requirements of mouse and human Th17 cells showed serum to be a confounder influencing the outcome of T cell polarization. Initially, it was claimed that these requirements differ between the two species and that for human Th17 differentiation TGF-β would be a dispensable factor [58], [59]. However, follow-up experiments in serum-free medium revealed that TGF-β is also required for human Th17 polarization and that the serum was probably contaminated with platelets that are a source of TGF-β [60]. Since serum is a considerable source of artefacts due to lot-to-lot variability and variable protein contents, we established the assay in serum-free AIM-V medium.

As a proof of concept that our assay can be applied to address biological questions, we have shown that DC triggered with various bacterial compounds have different capacities to induce Th1 polarization. This illustrates that, indeed, the environmental DC instruction influences the fate of the CD4^+^ T cells. We demonstrated a profound difference in the Th1 polarizing capacity of different bacterial fragments, with LPS being at least 100-fold less potent to induce IFN-γ secretion compared with the bacterial lysates FMKp and HKLM. Importantly, we also showed qualitative differences in capacity of different DC subsets to induce Th1 responses upon a particular PRR stimulus. Whereas LPS maturation of moDC did not result in high IFN-γ secretion in naive T cells, LPS-triggered pDC did respond with a pronounced transcriptional induction of T-bet and IFN-γ and the secretion of IFN-γ by T cells after 7 days of co-culture. Moreover, we showed that PGE_2_/TNFα-matured moDC did not lead to Th1 induction but instead to Th2 cells. This finding is consistent with previous studies [3], [61]. In contrast to de Jong et al. [4], we showed that this Th2 induction was independent of IL-4, as we neither detected IL-4 mRNA levels nor the secretion of the protein. Findings of other groups did show the possibility for IL-4 independent Th2 induction by pDC [62].

Complementary APC-independent tools, mimicking the APC by replacing it e.g. by anti-CD3 (± anti-CD28) or by feeder cells, represent a good approach to study the involvement of soluble DC-derived factors. However, anti-CD3 and anti-CD28 exert polarizing capacities on their own; this may vary depending on which clones of anti-CD3 or anti-CD28 antibodies are used. It has been shown that triggering of murine CD4^+^ T cells, *in vitro* and *in vivo*, by anti-CD28 led to the induction of GATA3 and to the secretion of Th2-specifying cytokines [63]. Additionally, the presence as well as the strength of TCR-signaling influences the fate of a naive CD4^+^ T cells independently of the cytokine milieu [64]–[66]. Strong TCR-signaling induces Th1 cells, whereas weak TCR-stimulation favors Th2 responses [67]. We tried to titrate down anti-CD3 to such an extent that it resulted in similar proliferative responses as observed in the co-culture experiments. We also observed that high concentrations of immobilized anti-CD3 induced the polarization of naive CD4^+^ T cells in the absence of polarizing signals.

We propose a new combination of tools to study human DC-induced naive CD4^+^ T cell polarization in a small-scale, autologous set-up. The assay combines analyses on transcriptional and protein levels. It allows comparing the magnitude, kinetics, and direction of a naive CD4^+^ T cell response after interaction with different PRR-experienced DC. The combination of monitoring simultaneously the expression kinetics of the different Th subset-related transcription factors and their cytokine release in one culture condition offers new perspectives on studying the priming phase of CD4^+^ T cells. The continuous monitoring of transcription factor expression over time allows studying differences in the kinetics of transcriptional induction by different DC and identifying whether a transient expression of transcription factors is responsible for functional subset specialization. Furthermore, this system can be of great value to investigate the influence of immunomodulating drugs or environmental factors on the initiation of human T cell responses and Th cell fates. The most effective moments of drug administration can be revealed and factors on which they eventually have a direct influence may be identified. In addition, it is possible to study epigenetic changes related to the priming phase of a T cell response, in an APC-dependent manner, such as the regulation of Th-specific enhancers.

Overall, this standardized assay will allow a more straightforward comparison of results obtained by different groups and will help to further extend our knowledge on the initiation of human naive CD4^+^ T cell responses.

## Supporting Information

Figure S1 **Optimization of CD4^+^CD45RA^+^ T cell isolation using immunomagnetic beads.** PBMC were isolated from whole blood by density centrifugation. CD4^+^CD45RA^+^ T cells were isolated by negative immunomagnetic separation according to the manufacturer’s instructions. To achieve highly pure naive CD4^+^ T cells, CD4^+^CD45RA^+^ T cells were purified a second time. The purification protocol was optimized by using the flow-through from the first separation followed by another round over a column, incubation with beads and purification over a second column or incubating with antibody-mix and beads and running it over a column. Purity staining of PBMC, first separation of CD4^+^CD45RA^+^ T cells and of different second separations of CD4^+^CD45RA^+^ cell populations were performed and analyzed by flow cytometry. Cells were gated on lymphocytes in FSC/SSC and on the living cells (7-AAD negative) and set as 100%. CD4^+^ T cells were gated on CD3^+^/CD4^+^ cells and furthermore discriminated between CD45RO^+^ and CD45RA^+^. Percentages of the different populations are indicated in the dot plots.
(TIF)

Figure S2 **Optimization of DC:T cell ratios.** 24 h-matured FMKp/IFN-γ DC were washed and added at different concentrations to a round 96-well plate: 1×10^4^ (light gray circle), 2×10^4^ (dark gray square) or 5×10^4^ (black triangle) and co-cultured with 5×10^4^ naive CD4^+^ T cells for 7 days in the presence of 24 h-FMKp/IFN-γ-matured DC-derived supernatant. Transcriptional induction of T-bet and IFN-γ as well as secretion of IFN-γ were determined. Data shown are representative of 4 independent experiments.
(TIF)

Figure S3 **Purities of differently isolated CD4^+^ T cell populations.** (**A**) Purity staining of total CD4^+^, CD4^+^CD45RA^+^, and CD4^+^CD45RO^+^ T cells after negative immunomagnetic isolation from freshly isolated PBMC. Percentage of CD3^+^ cells is expressed as percentage of total living singlet cells. Percentages of CD4^+^ cells are expressed related to total CD3^+^ cells and those of CD45RA^+^ and CD45RO^+^ cells are related to CD4^+^ T cell population. (**B**) Increasing percentages (0–10%) of CD45RO^+^ contamination into pure CD4^+^CD45RA^+^ T cell population.
(TIF)

Table S1 **Primers for Th lineage-specifying transcription factors used by real-time PCR.**
(DOCX)
