# Supplementary figures and images for: Monitoring the Initiation and Kinetics of Human Dendritic Cell-Induced Polarization of Autologous Naive CD4+ T Cells
Source: PLoS One. 2014 Aug 21;9(8):e103725. doi: 10.1371/journal.pone.0103725 (PMC4140687; doi:10.1371/journal.pone.0103725)

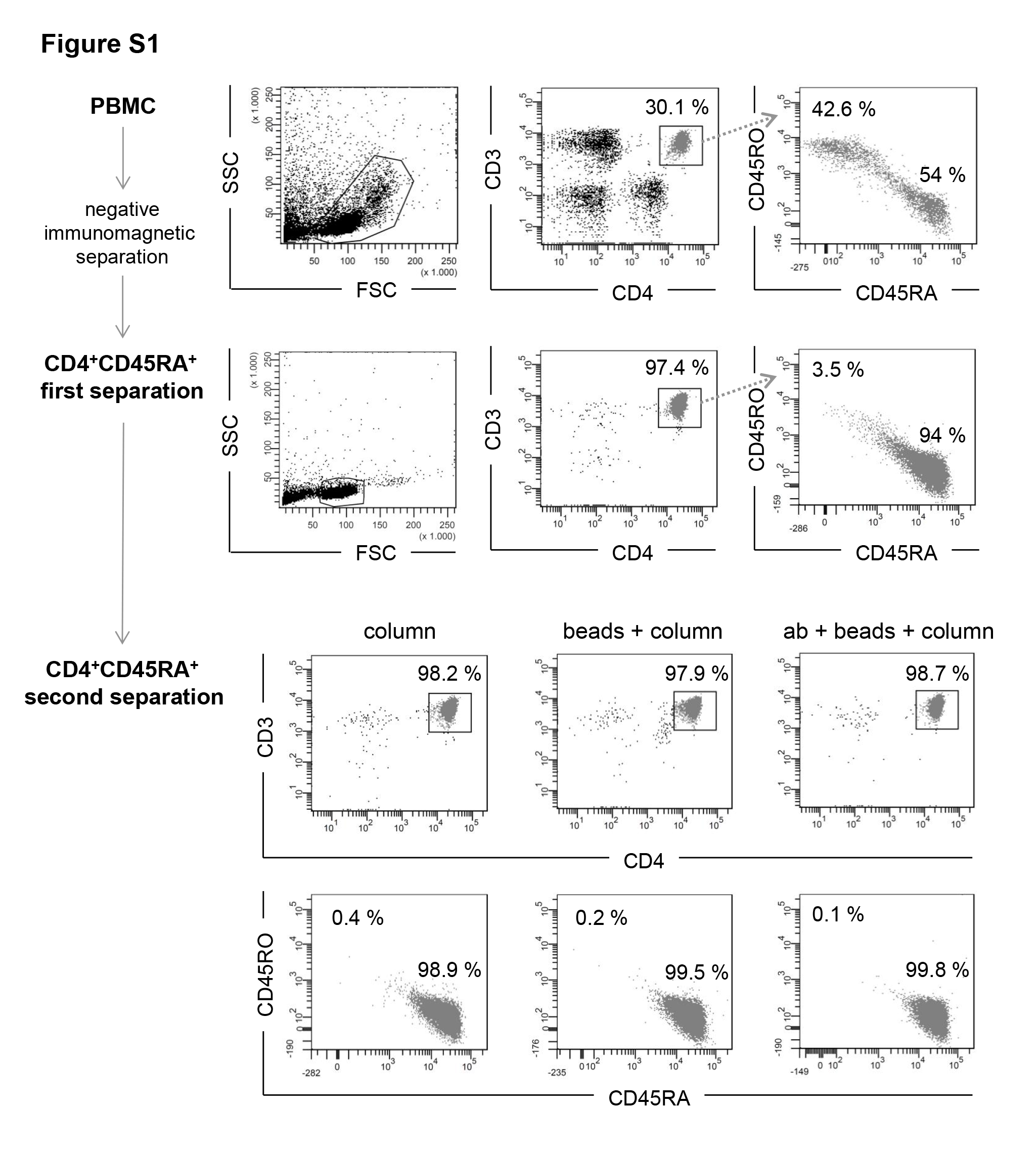

Supplement: Figure S1 — Optimization of CD4+CD45RA+ T cell isolation using immunomagnetic beads. PBMC were isolated from whole blood by density centrifugation. CD4+CD45RA+ T cells were isolated by negative immunomagnetic separation according to the manufacturer’s instructions. To achieve highly pure naive CD4+ T cells, CD4+CD45RA+ T cells were purified a second time. The purification protocol was optimized by using the flow-through from the first separation followed by another round over a column, incubation with beads and purification over a second column or incubating with antibody-mix and beads and running it over a column. Purity staining of PBMC, first separation of CD4+CD45RA+ T cells and of different second separations of CD4+CD45RA+ cell populations were performed and analyzed by flow cytometry. Cells were gated on lymphocytes in FSC/SSC and on the living cells (7-AAD negative) and set as 100%. CD4+ T cells were gated on CD3+/CD4+ cells and furthermore discriminated between CD45RO+ and CD45RA+. Percentages of the different populations are indicated in the dot plots. (TIF) [file pone.0103725.s001.tif]

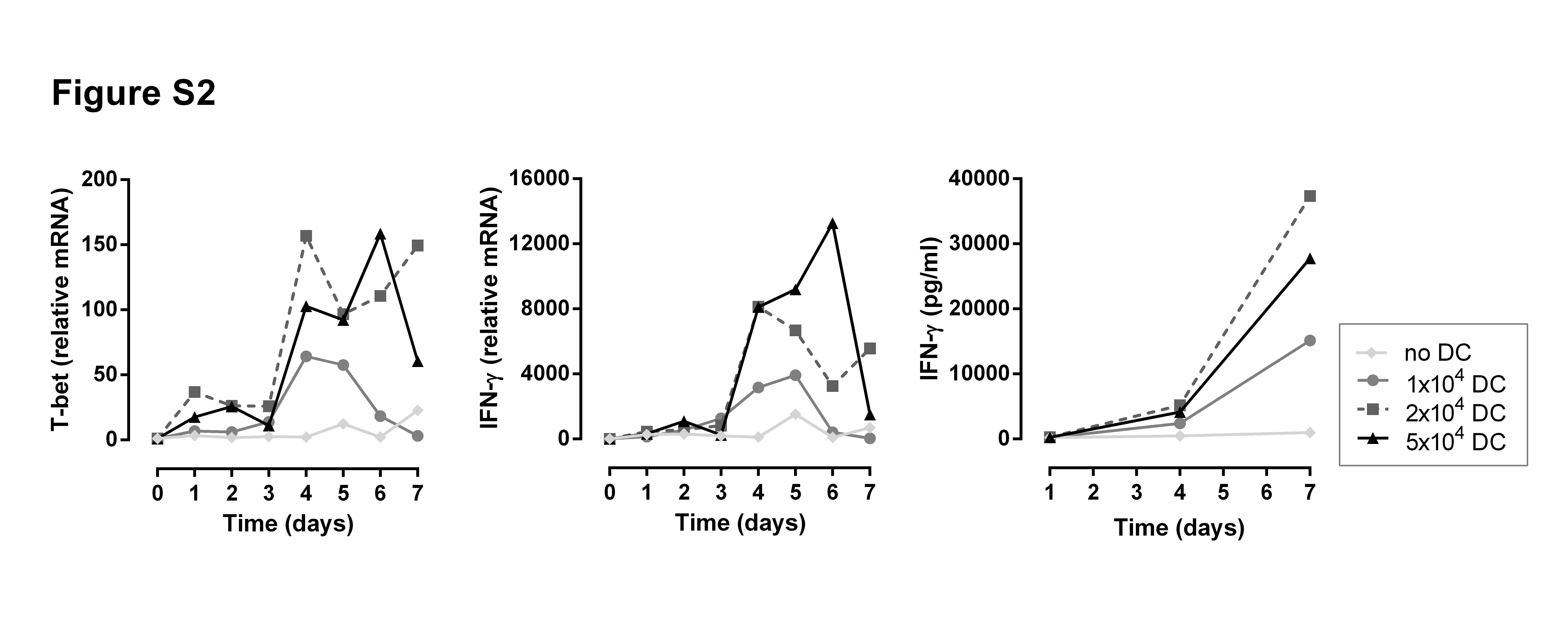

Supplement: Figure S2 — Optimization of DC:T cell ratios. 24 h-matured FMKp/IFN-γ DC were washed and added at different concentrations to a round 96-well plate: 1×10^4^ (light gray circle), 2×10^4^ (dark gray square) or 5×10^4^ (black triangle) and co-cultured with 5×10^4^ naive CD4+ T cells for 7 days in the presence of 24 h-FMKp/IFN-γ-matured DC-derived supernatant. Transcriptional induction of T-bet and IFN-γ as well as secretion of IFN-γ were determined. Data shown are representative of 4 independent experiments. (TIF) [file pone.0103725.s002.tif]

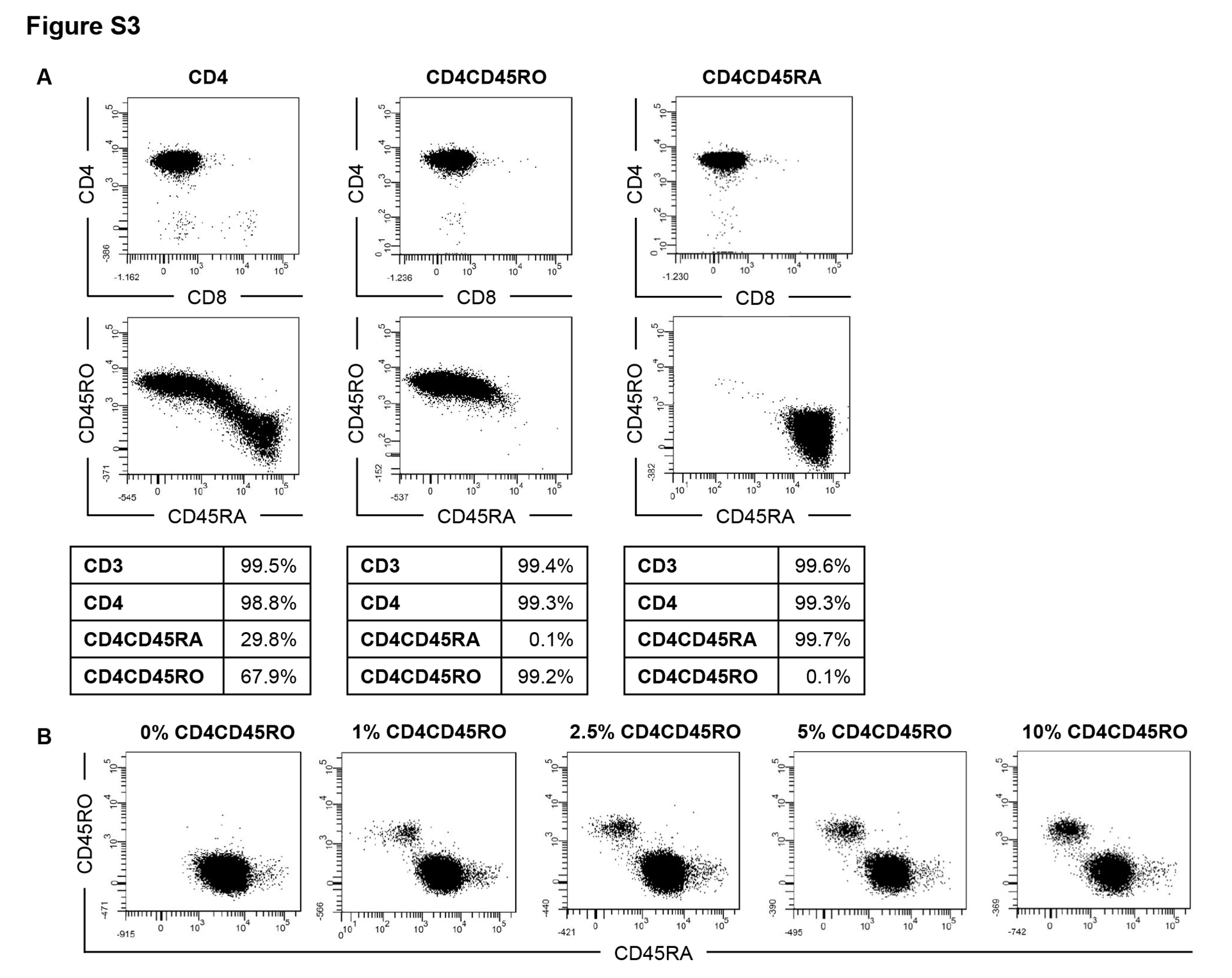

Supplement: Figure S3 — Purities of differently isolated CD4+ T cell populations. (A) Purity staining of total CD4+, CD4+CD45RA+, and CD4+CD45RO+ T cells after negative immunomagnetic isolation from freshly isolated PBMC. Percentage of CD3+ cells is expressed as percentage of total living singlet cells. Percentages of CD4+ cells are expressed related to total CD3+ cells and those of CD45RA+ and CD45RO+ cells are related to CD4+ T cell population. (B) Increasing percentages (0–10%) of CD45RO+ contamination into pure CD4+CD45RA+ T cell population. (TIF) [file pone.0103725.s003.tif]
