# Supplementary material for: Monitoring the Initiation and Kinetics of Human Dendritic Cell-Induced Polarization of Autologous Naive CD4+ T Cells
Source: PLoS One. 2014 Aug 21;9(8):e103725. doi: 10.1371/journal.pone.0103725 (PMC4140687; doi:10.1371/journal.pone.0103725)
Supplement: Table S1 — Primers for Th lineage-specifying transcription factors used by real-time PCR. (DOCX) [file pone.0103725.s004.docx]

**Table S1. Primers for Th lineage-specifying transcription factors used by real-time PCR.**

| **Gene** | **1) forward primer 2) reverse primer** | **amplicon length (bp)** | **Ta (°C)** | **melting curve** | **amplified transcripts** |
| --- | --- | --- | --- | --- | --- |
| CD3E | 1) TGA-GGG-CAA-GAG-TGT-GTG-AG | 223 | 58°C | 80-90°C | CD3E-001, CD3E-007, CD3E-009 |
|  | 2) TAG-TCT-GGG-TTG-GGA-ACA-GG |  |  |  |  |
| FOXP3 | 1) GAA-ACA-GCA-CAT-TCC-CAG-AGT-TC | 100 | 58°C | 77-86°C | FOXP3-001, FOXP3-002, FOXP3-003, FOXP3-004, FOXP3-201, FOXP3-202 |
|  | 2) ATG-GCC-CAG-CGG-ATG-AG |  |  |  |  |
| GATA3 | 1) ACT-ACG-GAA-ACT-CGG-TCA-GG | 179 | 58°C | 84-92°C | GATA3-001, GATA3-002 |
|  | 2) GAT-GGA-CGT-CTT-GGA-GAA-GG |  |  |  |  |
| IFNG | 1) GAA-GAA-TTG-GAA-AGA-GGA-GAG-TGA | 219 | 62°C | 74-83°C | IFNG-001 |
|  | 2) TGG-ACA-TTC-AAG-TCA-GTT-ACC-G |  |  |  |  |
| IL4 | 1) GCC-ACC-ATG-AGA-AGG-ACA-CT | 152 | 59°C | 80-90°C | IL4-001, IL4-002 |
|  | 2) ACT-CTG-GTT-GGC-TTC-CTT-CA |  |  |  |  |
| IL5 | 1) CAA-ACG-CAG-AAC-GTT-TCA-GA | 273 | 58°C | 78-86°C | IL5-001, IL5-003 |
|  | 2) CAG-TAC-CCC-CTT-GCA-CAG-TT |  |  |  |  |
| IL10 | 1) TCC-CTG-TGA-AAA-CAA-GAG-CA | 170 | 56°C | 75-84°C | IL10-001, IL10-002, IL10-003 |
|  | 2) ATA-GAG-TCG-CCA-CCC-TGA-TG |  |  |  |  |
| IL13 | 1) CAT-GGC-GCT-TTT-GTT-GAC-CA | 181 | 61°C | 79-90°C | IL13-001 |
|  | 2) AGC-TGT-CAG-GTT-GAT-GCT-CC |  |  |  |  |
| IL17A | 1) ACC-AAT-CCC-AAA-AGG-TCC-TC | 171 | 62°C | 78-87°C | IL17A-001 |
|  | 2) GGG-GAC-AGA-GTT-CAT-GTG-GT |  |  |  |  |
| IL17F | 1) TGA-AGC-TTG-ACA-TTG-GCA-TC | 174 | 55°C | 78-87°C | IL17F-001, IL17F-002 |
|  | 2) TTC-CTT-GAG-CAT-TGA-TGC-AG |  |  |  |  |
| RORC | 1) GGG-ATC-CAC-TAC-GGG-GTT-AT | 285 | 62°C | 84-91°C | RORC-001, RORC-002, RORC-201 |
|  | 2) GAC-CAC-TGG-TTC-CTG-TTG-CT |  |  |  |  |
| TBX21 | 1) CCG-TGA-CTG-CCT-ACC-AGA-AT | 158 | 58°C | 77-87°C | TBX21-001 |
|  | 2) ATC-TCC-CCC-AAG-GAA-TTG-AC |  |  |  |  |
| TGFB1 | 1) GGG-ACT-ATC-CAC-CTG-CAA-GA | 239 | 62°C | 87-95°C | TGFB1-001 |
|  | 2) CCT-CCT-TGG-CGT-AGT-AGT-CG |  |  |  |  |
